# Obstructive oligo-anuria revealing pelvic gynecological cancers, analysis of a series of 102 cases

**DOI:** 10.1016/j.amsu.2022.103332

**Published:** 2022-02-09

**Authors:** A. Kbirou, M. Sayah, F. Sounni, M. Zamd, M.G. Benghanem, M. Dakir, A. Debbagh, R. Aboutaib

**Affiliations:** a Urology Department, Ibn Rochd University Hospital Center, Casablanca, Morocco; b Department of Nephrology, hemodialysis and kidney transplantation, Ibn Rochd University Hospital Center, CASABLANCA, Morocco; c Faculty of Medicine and Pharmacy of Casablanca, Hassan II University, Casablanca, Morocco

**Keywords:** Obstructive olio-anuria, Pelvic gynecological cancers, Hemodialysis, Percutaneous nephrostomy

## Abstract

**Introduction:**

Obstructive Anuria (OA) constitutes a diagnostic and therapeutic emergency involving the vital prognosis of the patient requiring an emergency and multidisciplinary care. The etiologies are multiple and the pelvic gynecological cancers represent one of the main causes of OA.

**Objectives:**

Describing the epidemiological, clinical, paraclinical, etiological, therapeutic and progressive aspects of obstructive anuria of the gynecological origin, in the urology department in the University Hospital Center.

**Materials and methods:**

This is a descriptive and retrospective study spread over a period of 4 years (2016–2019) including all the patients admitted for management of OA secondary to the pelvic gynecological cancers.

**Results:**

102 patients were included in the study whose the mean age was 60 years old (36–84). The main etiologies were cervical cancer (93%), followed by endometrial cancer (5%) and ovarian cancer (2%). The mean time to consultation was 4.5 days (1–8). The main circumstances of discovery were anuria (67%), oligoanuria (21.5%), low back pain (17%) and hematuria (9%). Clinical examination found an altered general condition (Performans Status> 2) in 37.5% of the patients and an advanced local state in 96% of the patients. The means of serum creatinine and blood urea were 122 mg/l and 2.4 g/l respectively. The hemodialysis (HD) was indicated in 29.5% of patients with life-threatening hyperkalemia (Kalemia> 6.4meq/l) with cardiac distress (20.5%), hydro-sodium overload (6%) and metabolic acidosis (3%). The ultrasound-guided percutaneous nephrostomy was the main method of diversion (92%) followed by the placement of the double J stent (8%). The outcome was favorable in the majority of patients with normalization of the kidney function (88%) while 7% of cases kept chronic kidney disease. The main complication was an obstruction syndrome (41%), followed by infections of the percutaneous nephrostomy tubes (13%) and venous thrombosis of the lower limbs (3%). In addition, the mortality was estimated at 5%.

**Conclusion:**

The obstructive anuria constitutes a medico-surgical emergency involving the patient's vital prognosis. Our study notes the frequent association between the pelvic gynecological tumors and the obstructive anuria, which can be explained by the advanced stage of these tumors. This work underlines the fundamental interest of early diagnosis of these tumors to enable the prevention of the OA.

## Highlights

• Obstructive anuria is a diagnostic and therapeutic emergency.
• Obtructive oligo-anuria presents a frequent mode of revelation of pelvic gynecological cancers.
• Obstructive anuria is in favor of the locally advanced stage of pelvic gynecological tumors.
• The prevention is based on screening for these cancers allowing early diagnosis.

## 1 Introduction

Anuria is defined by a total cessation of diuresis or by a volume less than 200, or even less than 400 ml/24 h according to the authors [1]. It constitutes a medico-surgical emergency involving the patient's vital prognosis because of the rapid onset of acute kidney disease (AKI), requiring emergency management in a specialized unit.

Obstructive or post-renal anuria (10% of the anurias), is related to an obstruction located at any level of the upper excretory tract, including the ureteral meatus [1,2]. The obstacle, intrinsic or extrinsic, can be double and sit on the excretory route of both kidneys or unique and sit on the excretory route of a single anatomical or functional kidney [1,2]. The etiologies are multiple; in 50% of the cases the origin of the anuria is neoplasic [1]. In women, the closeness anatomic relation between the pelvic organs (the cervix, the ovary …) and the ureter explains the OA's frequency due to compression by pelvic gynecologic cancers (PGC).

Faced with any obstructive anuria in a woman, we have to seek a pelvic tumor. Generally in developing countries and particularly in Morocco, there was a little work studying this frequent association between OA and PGC. On this subject, a study conducted in 107 cases of OA found that PGC was the responsible in 39% of the cases [3].

In Africa, a study carried out in Uganda over a period of 6 months in 283 women followed for cervical cancer of the uterus confirmed at the advanced stage, revealed that the obstructive anuria was a frequent complication of these types of cancers, ie 39% of these patients [4]. The objective of our study was to describe the epidemiological, clinical, paraclinical, etiological, therapeutic and evolutionary aspects of OA of PGC's origin, a study conducted in the urology department of University Hospital Center. This study has been reported according to SCARE 2020 criteria [5].

## 2 Materials and methods

This is a descriptive and retrospective study spread over a period of 4 years from January 1, 2016 to December 31, 2019 including all the patients admitted for management of OA secondary to the pelvic gynecological cancers (cervical cancers, ovary and endometrium). This study registered at http://www.researchregistry.com Research Registry UIN: researchregistry 7226.

We excluded the patients already followed for chronic kidney disease and presenting a PGC and patients with an acute kidney injury secondary to nephrotoxic drugs such as chemotherapy.

The quantitative and qualitative variables concerned epidemiology (number of cases, age), clinic (clinical signs and examination), paraclinic (biological and radiological), therapy (medical, surgical and hemodialysis) and evolution, shown in Table 1.

**Table 1:** The variables studied.

Epidemiological	Number, Age,	
Clinics	Clinical signs	Anuria, oligoanuria, low back pain, hematuria
	Clinical examination data	General examination, Pelvic examination, speculum examination
Paraclinics	Radiology	Renovesical ultrasound, Abdomino-pelvic scanner
	Biology	Blood count, electrolyte balance
Therapeutic	Therapeutic methods	Medical treatment, Hemodialysis, Type of diversion
Evolution	Favorable, chronic kidney disease, mortality	

Before any operative procedure, the patients were hospitalized in the surgical intense care unit for hemodynamic stabilization by the correction of the hypovolemia (transfusion and hydration) and the correction of serious metabolic disorders secondary to OA by hemodialysis, especially in the presence of signs of heart disease and metabolic acidosis.

The antibiotics were systematic before surgical diversion to prevent septic discharge.

During the data collection, we insisted on the respect of the ethical rules of the University Hospital Center's ethic committee through taking the patient's consent, keeping the confidentiality of the data collected and respecting the anonymity of the patients concerning the OA and the underlying pathology. The discontinuous values were expressed by numbers and percentages and were compared with a “Chi2” test. The differences were considered significant for a “p” < 0,05.

## 3 Results

**Fig. 1:**
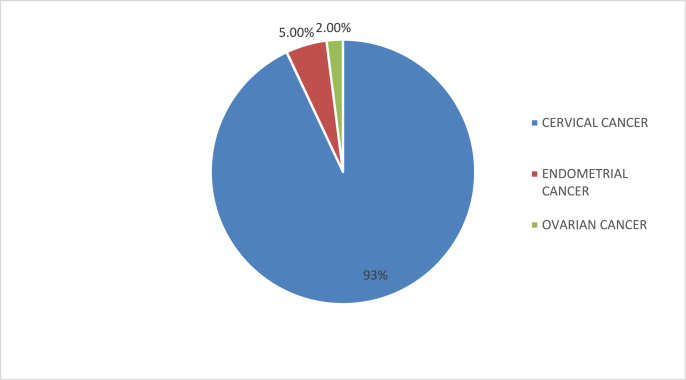
The main pelvic gynecological cancers responsible for the OA.

102 patients were included in the study whose the mean age was 60 years old with extremes of 36 and 84 years old. The main pelvic gynecological cancer responsible for an OA was the cervical cancer found in 95 patients (93%), followed by the endometrial cancer found in 5 patients (5%) and the ovarian cancer in 2 of our patients (2%) [Fig. 1].

The average consultation time was 4.5 days with extremes of 1 and 8 days. The main circumstances of discovery were the anuria found in 68 cases (67%) (P < 0.019), the oligoanuria that was found in 22 cases (21.5%) (P < 0.045), low back pain in 19 patients (17%) (0, 0.217) and hematuria in 9 patients (9%) (0.051). Clinically, 38 patients (37.5%) had an altered general condition (Performans Status> 2) and all the rest of the patients, i.e. 64 cases (62.5%) were in a good general condition (Performans Status = 0/1).

Regarding the local condition, the pelvic examinations (the vaginal examination coupled with the rectal examination) found infiltrated parameters in 98 cases, i.e. 96% of patients (P < 0.038) and flexible parameters in 4 of our patients (4%) (P < 0.045).

**Fig. 2:**
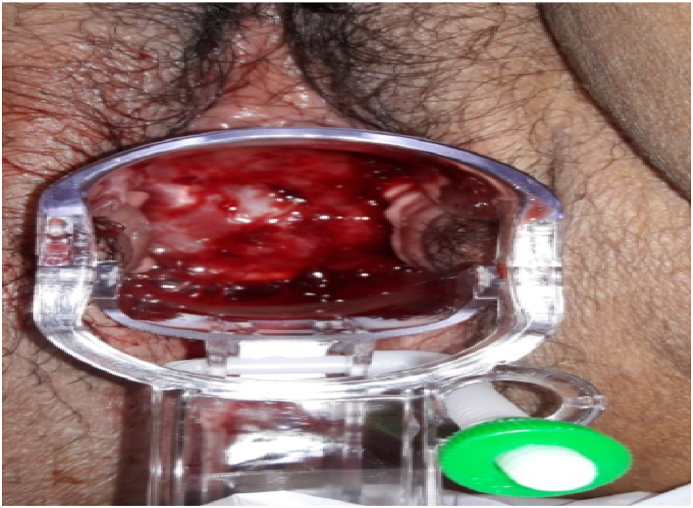
Speculum examination demonstrating an ulcerative uterine cervical lesion in favor of a cervical cancer revealed by an obstructive anuria in a 47-year-old patient.

The speculum examination found lesions in the uterine cervix in 96 patients (94%) and did not reveal any lesion in the other cases, i.e 6 cases (6%) (Fig. 2). The bladder catheterization was performed in all the patients with an average diuresis of 125 ml (50–200) (P < 0.028).

The blood electrolyte balance sheet including mainly creatine, urea, potassium and alkaline reserves found mean levels of creatinemia and blood urea of 122 (42–202) mg/l (P < 0.017) and 2.4 (0.8–4) g/l (P < 0.015) respectively. The serum potassium was increased in 52 patients (62.5%) (P < 0.128) and normal in 38 patients (37.5%) (P < 0.254).

Reno-bladder ultrasound found dilation of the renal cavities in the majority of the cases i.e in 97 patients (95%) (P < 0.042) while 5 patients (5%) (P < 0.051) did not present a dilation; moreover the bladder was semi-full on the ultrasound in 85 patients (83.5%) and empty in 17 patients (6.5%).

**Fig. 3:**
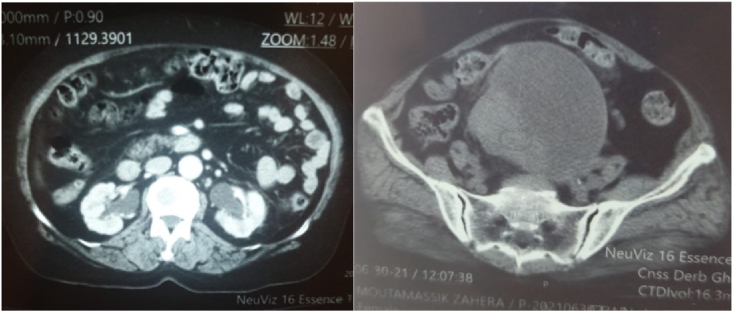
A scannographic appearance in favor of a solidocystic ovarian tumor mass with inguinal lymphadenopathy and hydronephrosis.

The pair of the abdominopelvic non-contrast computed tomography scan and the magnetic resonance imaging played an essential role in the local evaluation of these tumors, thus allowing to stage these tumors. For cervical cancers, imaging confirmed the locally advanced stage of the patients, thus we distinguished 64 patients (67.5%) (p < 0.071) that were at the stage IV and 29 patients (32, 5%) (p < 0.076) that were in the stage III, according to the FIGO classification. In addition, in the radiology images that objectified the large latero-uterine masses in favor of the ovarian origin, the average dimension was 19.5 cm (11–28) [Fig. 3].

Regarding the therapeutic methods, the medical treatment based on calcium gluconate and insulin was indicated in all the patients with hyperkalemia with or without cardiac suffering, while awaiting hemodialysis due to the reduced number of the hemodialysis's “devices” available in our context.

The emergency HD was indicated in 30 patients (29.5%) (P < 0.213) whose the main indications were life-threatening hyperkalemia (Kalemia> 6.4meq/l) with cardiac distress in 21 patients (20, 5%) (P < 0.145), the hydrosodic overload in 6 cases (6%) (P < 0.217) and a metabolic acidosis in 3 patients (3%) (P < 0.012). The intermittent HD was indicated in 23 patients (22.5%) until the improvement of the kidney functions with a mean duration of 6.5 days (2–11).

**Fig. 4:**
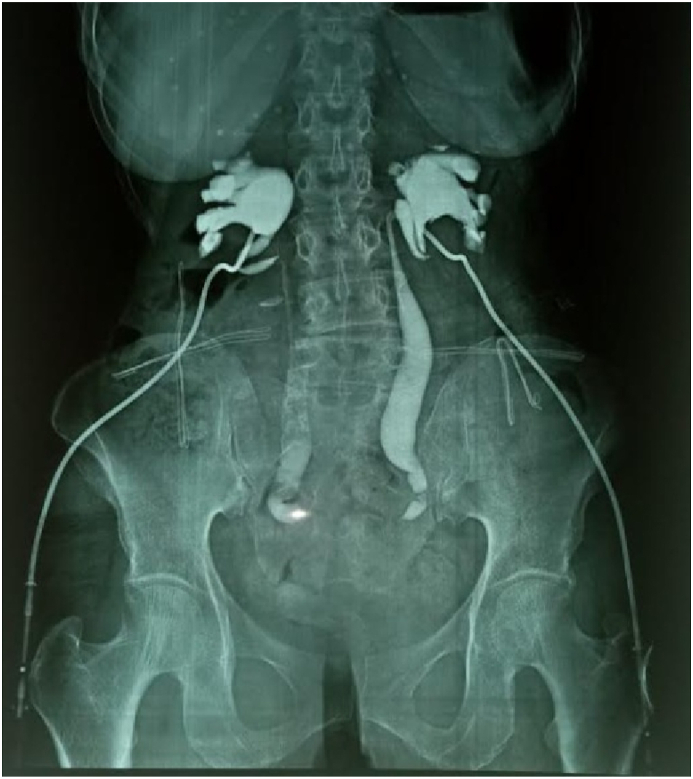
A descending pyelography in a patient admitted for OA secondary to cervical cancer derived by bilateral percutaneous nephrostomy showing a clear stop in the passage of the contrast agent to the pelvic level making it impossible to raise the double-J stent.

The ultrasound-guided percutaneous nephrostomy was the main method of the derivation of the upper urinary tract in 94 cases (92%) (P < 0.02) followed by the placement of the double J stent in 8 patients (8%) (P < 0.07) (Fig. 4). The evolution after the bypass was favorable in the majority of the patients with a normalization of the kidney function in 90 patients (88%) while 7 patients, i.e 7% of the cases kept a chronic kidney disease and underwent to a permanent HD. In addition, the mortality was estimated at 5%.

The surveillance objectified as a main complication the obstruction syndrome that was observed in 42 cases (41%), followed by the percutaneous nephrostomy tube's infections in 13 patients (13%) and the venous thrombosis of the lower limbs in 3 patients (3%).

After the patients's stabilization, we proceeded to the diagnosis confirmation with the histology; and revealed epidermoid carcinoma as the main histological type of cervical cancer in 93 of the patients (91%), followed by the adenocarcinoma in 2 patients (2%). The endometrioid adenocarcinoma was found in all the patients with endometrial cancer (5%). About the ovarian masses, the ovarian sarcoma and the serous cystadenoma (2%) were individualized.

## 4 Discussion

This study objectified an important way of revelation of the pelvic gynecological cancers, the OA, which can be serious and responsible for significant mortality and complications of the management of this type of tumors which are generally locally advanced; in the literature there were few studies dealing with this frequent association especially in developing countries.

Obstructive anuria is a frequent cause of the AKI and represents 5–10% of the cases [6] and 22% of the cases of the AKI in the elderly population [7]. It constitutes a diagnostic and a therapeutic emergency involving the patient's vital prognosis and requiring multidisciplinary management. The OA's etiologies are multiple and the neoplasic causes constitute one of the main etiologies in approximately 50% of the cases [1]. The pelvic gynecologic tumors are a common cause of the OA. Efesoy and al, revealed in a series of 347 cases that cervical cancer was responsible of the OA in 16% of the cases followed by the endometrial cancer (1.4%) and the ovarian cancer (1.4%) [8]. However, Romero and al. reported the cervical cancer (CC) as the main etiology of the obstructive urinary tract tumors (53.5%) followed by the endometrial cancer (7%) [9]. Liang and al reported that CC (43%) is the main cause, followed by the ovarian cancer (29%) and the endometrial cancer (25%) [10]. Our study revealed the frequent association between the pelvic gynecological cancers and the OA due to the advanced stage of these tumors. The advanced stage was explained by the diagnosis's delay, and especially the lack of screening but also the use of the traditional medicine represented by self-medication by plants in addition to their ineffectiveness, these plants could have a harmful effects on the kidney functions aggravating thus the prognosis of the OA in these patients [11]. The age is a decisive prognosis factor in the management of these patients, the average age of our patients was 60 years old as well as Liang's but higher than the Efesoy's [11,12].

The diagnosis is evoked clinically by the absence of the diuresis for at least 24 h, sometimes it's difficult if there is a residual diuresis manifested by an oliguria [1]. Other signs may be present or linked to the locoregional invasion of the PGC such as a low back pain and an hematuria or the related signs to the tumor such as the menometrorrhagia, the abdominal pain or the abdominal distention. The anuria was the main clinical manifestation (67%). While Mabrouk et Al. reported the oliguria as the main manifestation (71.96%) [2], Atuhairwe et Al. found OA as the clinical sign in 39.6% of the cases [3].

Certain clinical forms can be deceptive in the presence of non-specific signs related to the AKI and depending on its depth and the speed of its onset, these manifestations can be digestive (vomiting, nausea and hiccups) [12], neurological (disturbances of consciousness) and respiratory (dyspnea and orthopnea) [13]. These were not only signs of seriousness but could also have had an impact on the general condition, which explained the deterioration of the general condition of our patients [14].

The biological assessment essentially included the blood ionogram objectifying an increase in the blood levels of creatine and urea, and highlighting serious biological elements such as a life-threatening hyperkalemia and metabolic acidosis requiring urgent extrarenal purification [13].

The first radiological assessment to be requested is the abdominopelvic ultrasound which constitutes a reliable examination to confirm or eliminate an obstacle of the excretory tract showing a bilateral or unilateral dilation on a single kidney of the pyelocalicular cavities, associated with an empty or poorly filled bladder, which is in favor of the obstructive origin of anuria [16]. In our series, dilation was demonstrated in 95% of the cases while Atuhairwe and Al. and Misra and Al. reported dilation in 47% [4] and 77% of cases respectively [17].

In certain situations, the absence of dilation of the renal cavities could leave a doubt on the obstructive origin especially in front of the neoplastic antecedents [18]. Ultrasound informs hardly about the nature and the seat of the neoplastic obstacle but allows to evaluate the renal cavities for a possible derivation by a percutaneous nephrostomy [15].

Once the diagnosis of the OA is established, the hospitalization in an intensive care unit is essential due to the seriousness of the clinical picture [18]. The basic treatment is the removal of the obstacle but the medical management is fundamental to correct the metabolic disorders such as an hyperkalemia without cardiac suffering, and to treat an infectious syndrome, an hemodynamic instability or a specific condition of the patient that is likely to worsen the course (cardiac insufficiency, pre-existing chronic kidney disease, hyperkalemic treatments particularly), however this should not delay the derivation of the upper urinary tractus [1].

In emergency, the extrarenal replacement therapy constitutes a saving and effective solution against the serious metabolic disorders (metabolic acidosis, hydrosodic overload) and a heart's protection against the life-threatening hyperkalemia [15]. Due to the PGC's blood loss associated, extra-renal purification allows us to avoid the overload's accidents by transfusing the patients on per dialysis in certain serious situations [19]. The HD was indicated in 30% of the cases with severe hyperkalemia as the main indication (20.5%), which was similar to the series by Mabrouk et al. which reported the life-threatening hyperkalemia as the main indication [3].

The urinary drainage is performed either retrogradely (single ureteral catheter or double J catheter) [1] or by the percutaneous nephrostomy [7]. The percutaneous nephrostomy was the main method of the urine derivation due to the PGC's advanced stage evaluated clinically by the pelvic touches and radiologically by the descending retrograde pyelography which didn't allow us to install the double-J stent and unfortunately impaired the quality of life of our patients. However, Ganatra et al. Had reported on a series of 157 cases followed for a malignant extrinsic compression (uterus tumors (10%) and an ovarian tumors (17%)) requiring a bypass of the upper urinary tractus, a rate of failure of the attempt to raise the J probe (37.7%) with the use of percutaneous nephrostomy [20]. While all of Romero and Al. patients had have a derivation by percutaneous nephrostomy.

After the derivation, monitoring is essential to detect complications [21], the principal one is the obstacle removal syndrome treated by the compensation for hydro-electrolytic losses by the careful administration of fluids while paying attention to the risk of overload [22].

Despite the advent of the diagnostic and therapeutic methods, the OA is often associated with a significant mortality. In our series, the mortality was 5% lower than it is in the literature which is 34.6% [2] and 30% [9] respectively, which could be explained by the young age of our patients and the absence of associated defects and comorbidities.

## 5 Conclusion

Despite the advent of the diagnostic and the therapeutic methods, the pelvic gynecological cancers are a frequent cause of the OA especially in the developing countries as well as Morocco where this type of tumor is diagnosed late. This study reveals the fundamental interest of the early diagnosis of the PGC especially the cervix cancer by insisting on the cervical cancer screening and the therapeutic education of the health professionals.

## Funding

We don't have any financial sources for our research.

## Declaration of competing interest

All authors disclose any conflicts of interest.

## Provenance and peer review

Not commissioned, externally peer-reviewed.

## Patient confidentiality

The references, figures, tables and any acknowledgements include not any confidential identifying patient information, such as the name or date of birth of the patient.

## Ethical approval

The study committee of the chu ibn Rochd hospital center approves the favorable opinion to publish this work.

## Sources of funding

This study did not receive any sources of funding

## Author contribution

Dr. AK, Dr. MS, and Dr. FS analysed and performed the literature research, Pr. MD, Pr.AD and RA performed the examination and performed the scientific validation of the manuscript. Issam Jandou was the major contributors to the writing of the manuscript. All authors read and approved the manuscript.

## Registration of research studies

1. Name of the registry: research registry
2. Unique Identifying number or registration ID: researchregistry7226
3. Hyperlink to your specific registration (must be publicly accessible and will be checked): https://www.researchregistry.com/browse-the-registry#home/

## Guarantor

Dr KBIROU Adil.

## Consent

The consent to publish this information was obtained from study participants. We confirm that written proof of consent to publish study participants are available when requested and at any time.

## Declaration of competing interest

No Conflicts of interest.

## Abreviations list

OA: Obstructive Anuria
PGC: Pelvic Gynecological Cancers
HD: Hemodialysis
